# Target Tracking While Jamming by Airborne Radar for Low Probability of Detection

**DOI:** 10.3390/s18092903

**Published:** 2018-09-01

**Authors:** Fei Wang, Xin-Bo Cong, Chen-Guang Shi, Mathini Sellathurai

**Affiliations:** 1 Key Laboratory of Radar Imaging and Microwave Photonics, Ministry of Education, Nanjing University of Aeronautics and Astronautics, Nanjing 211106, China; m18551716091@163.com (X.-B.C.); scg_space@163.com (C.-G.S.); 2 Institute of Sensors, Signals and Systems, Heriot-Watt University, EH14 4AS Edinburgh, UK; M.Sellathurai@hw.ac.uk

**Keywords:** low probability of detection (LPD), tracking, jamming, passive detection system (PDS), constant false alarm rate (CFAR)

## Abstract

Although radiation power minimization is the most important method for an advanced stealth aircraft to achieve the low probability of detection (LPD) performance against the opposite passive detection system (PDS), it is not always effective when the performance of PDS is advanced. In a target tracking scenario, an interference tactic is proposed in this paper to keep the airborne radar in an LPD state. Firstly, this paper introduces the minimization radiation power design of airborne radar based on the distance between the radar and the target, and introduces the minimization radiation power design of the airborne jammer based on the predicted detection probability of the opposite PDS. Then, after consulting the most commonly used constant false alarm rate (CFAR) technologies in passive detection systems, including the cell average CFAR, the greatest of CFAR, the smallest of CFAR and the ordered statistic CFAR, this paper analyzes their relationships and points out the way of interference. Finally, based on the constraints, not only including the predicted detection probabilities of airborne radar and opposite PDS, respectively, but also including the time synchronization which is necessary to avoid the leaked interference power generated by airborne jammer jamming the airborne radar echoes from the target, this paper establishes a math model to minimize the total interference power of airborne jammer without interfering target tracking. Simulation results show that the proposed model is effective.

## 1. Introduction

The Low probability of intercept (LPI) technology is used to protect the airborne radar from the threat of the opposite passive detection system (PDS). Stove (2004) proposed that LPI should be divided into at least two levels, low probability of detection (LPD) and low probability of exploitation (LPE) [1]. For LPE, Fancey (2010) analyzed many LPI signals from various aspects, and then proposed an empirical index to evaluate their LPE performance [2]. Shu (2017) proposed an advanced pulse compression noise waveform that uses random amplitude and phase changes to avoid being exploited by the opposite PDS [3]. Compared with LPE, LPD is an efficient method to improve the LPI performance of active radiation sources. One of the most significant ways to improve LPD performance is to minimize the radiated power. For the airborne radar tracking and search process, LPD performance has received more and more attention, because it helps to spare the airborne radar from the serious threat from an opposite PDS.

**Figure 1:**
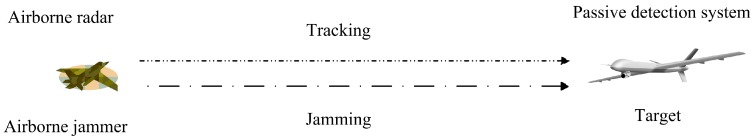
Combination of tracking and jamming at the target equipped advanced passive detection system.

In recent years, the LPD of airborne radar has been studied not only to study how to control its radiant power, but also to study how to control its irradiation interval and dwell time on target. In those documents, Krishnamurthy (2005) proposed a computationally efficient dynamic emission control and management algorithm to minimize the threat to the platform caused by the opposite PDS [4]. Liao (2011) proposed two radar radiation energy control strategies: minimum power strategy and minimum resident strategy [5]. Zhang (2011) proposed a radar search method that minimizes the radiant energy function as the optimization target, with the beam width, dwell time and average radiated power as optimization parameters. Simulation results show that the algorithm can not only ensure good detection performance, but also reduce energy consumption [6]. Liu (2015) proposed to minimize the intercept probability as an optimization target under a certain radar detection probability, which can be used to control radar radiant energy during the tracking process [7]. Andargoli (2015) provided a flexible and effective method to control radar power based on certain detection requirements [8]. She (2016, 2017) proposed a joint sensor selection and power allocation algorithm for multi-target tracking in radar networks based on LPD objectives, which helps to minimize the total transmit power of the radar network based on predetermined mutual information (MI) or the minimum mean-square error (MMSE) threshold between target impulse response and the reflected signal [9,10]. Zhang (2016) proposed a new radar network resource scheduling method for clutter tracking. Simulations show that compared with other methods, the LPD performance of this algorithm is better [11]. Zhang (2016) applied classical radar’s minimization radiation method to opportunistic array radars [12]. Simulations show that classical radar radiation control methods are also useful for opportunistic array radars. Shi (2016, 2017) presented a series of resource management methods for LPD and LPE in various contexts such as radar power, dwell time and illumination interval [13,14,15,16,17,18]. In addition to airborne radars, the minimization of interference power by jammers was studied to avoid positioning by anti-radiation system. Liu (2012) offered an interference power allocation model based on the detection probability in the active interference process [19]. Song (2014) proposed an adaptive control method of interference power aiming for LPD purpose by predicting radar echo power, which was useful for designing self-defense interference system [20]. Wang (2015) discussed the self-defense power method in the process of electronic countermeasures under LPD constraints [21]. Hao (2015) proposed a new interference method that can reduce the threshold detection probability and improve the interference power efficiency [22]. The serious threat to airborne radar comes from the advanced PDS developed in recent years. As far as we know, the constant false alarm rate is a key parameter of the existing PDS signal processing process. The most classical CFAR processing algorithm is the cell average constant false alarm (CA-CFAR) processing algorithm. However, CA-CFAR was analyzed in the Rayleigh clutter background and found that its detection performance was greatly influenced by the clutter characteristics. In order to reduce the impact of clutter in the tracking target process, a unit average maximum detector (Greatest of Constant False Alarm Rate, GO-CFAR) and a unit average selection detector (Smallest of Constant False, SO-CFAR)have been proposed [23,24,25]. To further improve the detection performance, luo (2009) and liu (2001) proposed a new CFAR detection algorithm which can not only process time domain data in, but also convert the data into frequency domain and wavelet domain for further processing [26,27]. He (2011) analyzed GO-CFAR, SO-CFAR and OS-CFAR. And results show that GO-CFAR can maintain a stable false alarm probability at the edge of the clutter, but the detection performance of GO-CFAR decreases in multi-target scenarios. On the contrary, SO-CFAR maintains good detection performance in multiple targets situations, but the false alarm capability at the edge of the clutter is seriously affected.The detection performance of OS-CFAR is between GO-CFAR and SO-CFAR [23,24,25]. An (2015) proposed a constant false alarm detection method based on summation. Compared with traditional detection methods, sum-CFAR can improve the detection probability of exponentially distributed clutter background [28]. Different from existing literatures, this paper comprehensively considers airborne radar and jammer and proposes a tracking while jamming tactic, which is helpful for airborne radar to maintain LPD state against advanced PDS. Section 2 describes the airborne radar’s adaptive minimum radiation power design criteria, which is based on the distance from the airborne radar to the target and the interception factor. Then, according to the probability of detection required by the radar receiver, this section shows that the combination of tracking while jamming in Figure 1 is an effective method to protect the airborne radar platform from the threat of the opposed advanced PDS. Section 3 first introduces several different CFAR, and then analyzes the detection probabilities in CA-CFAR, GO-CFAR, SO-CFAR and OS-CFAR, and proves that the minimum interference power design mainly depends on GO-CFAR, SO-CFAR, OS-CFAR and the number of reference cells. Section 4 establishes a math model to minimize the total interference power of airborne jammer without interfering target tracking based on the constraints of predicted detection probabilities and time synchronization.

## 2. Problem Scenario

The commonly used evaluation index of LPI performance of airborne radar is the interception factor proposed by Schleher, which is expressed as [29]:

(1) $$\alpha =\frac{R_{I\max}}{R_{R\max}}$$

where, $R_{I\max}$ is the maximum interception distance of the opposed PDS and $R_{R\max}$ is the maximum detection distance of the airborne radar.

(2) $$R_{R\max}^{4}=\frac{P_{t}G_{t}G_{rr}\sigma \lambda^{2}}{{\left(4\pi \right)}^{3}kT_{0}B_{r}F_{n}L\cdot SNR_{\min}}$$

where, $P_{t}$ is the radar radiated power; $G_{t}$ and $G_{rr}$ are antenna gains of the transmitter and the receiver respectively; $k=1.38\times 10^{-23}$ J/K is the Boltzmann constant; σ is the radar cross section(RCS) of the target; λ is the wavelength of the radar signal; $F_{n}$ is the noise coefficient; $T_{0}=290$ K is the standard noise temperature; $B_{r}$ is the bandwidth of the receiver; *L* is the loss of the radar system; $SNR_{\min}$ is the minimum detectable SNR.

(3) $$R_{I\max}^{2}=\frac{P_{t}G_{i}G_{ir}\lambda^{2}}{{\left(4\pi \right)}^{2}P_{i\min}}$$

where, $G_{ir}$ is the receiver antenna gain of the opposed PDS; $G_{i}$ is the transmission gain from the airborne radar to the opposed PDS; $P_{i\min}$ is the minimum detectable sensitivity of the opposed passive detection system. Taking (2) and (3) into (1), there is:

(4) $$\alpha ={\left[\frac{P_{t}G_{i}^{2}G_{ir}^{2}\lambda^{2}kT_{0}B_{r}F_{n}L\cdot SNR_{\min}}{4\pi P_{i\min}^{2}G_{t}G_{rr}\sigma }\right]}^{\frac{1}{4}}$$

Let $S_{I}=\frac{P_{i\min}}{G_{ir}}$ and $S_{R}=\frac{kT_{0}B_{r}F_{n}L\cdot SNR_{\min}}{G_{rr}}$, there is:

(5) $$\alpha ={\left(\frac{4\pi G_{i}S_{R}}{\sigma G_{t}S_{I}}\right)}^{\frac{1}{2}}R_{R\max}$$

During the target tracking process, the transmission gain in (3) is the same as the antenna gain in (2). Then, Equation (5) can be rewritten as:

(6) $$\alpha ={\left(4\pi \cdot \frac{S_{R}}{S_{I}}\cdot \frac{R_{R\max}^{2}}{\sigma }\right)}^{\frac{1}{2}}$$

From (1), it can be seen that if α>1, the opposed passive detection system can easily detect the airborne radar signal. If α < 1, the airborne radar signal may be in the LPI state. And if α = 1, combined with Formula (6), the critical detection distance is defined as:

(7) $$R_{c}={\left(\frac{\sigma }{4\pi }\cdot \frac{S_{I}}{S_{R}}\right)}^{\frac{1}{2}}$$

Then, according to Formula (2), the corresponding critical power is:

(8) $$P_{Rct}=\frac{{\left(4\pi \right)}^{3}R_{c}^{4}S_{R}}{\sigma \lambda^{2}G_{t}}$$

or according to Formula (3), the power is:

(9) $$P_{Rct}=\frac{{\left(4\pi \right)}^{2}R_{c}^{2}S_{I}}{\lambda^{2}G_{t}}$$

Substituing Formulas (8) and (9) into (2) and (3), without considering radar signal sorting and radar signal tracking processes by the opposed PDS, the airborne radar might be possible in the LPD state if the radiated power of the radar signal is:

(10) $$P_{Rct}\cdot {\left(\frac{R}{R_{c}}\right)}^{4}\leq P_{t}\leq P_{Rct}\cdot {\left(\frac{R}{R_{c}}\right)}^{2}$$

where, *R* is the distance from the airborne radar to the target. However, the solution of $P_{t}$ in (10) is not always available. The radiated power constraint in (10) is only for single pulsed radar signals. For pulse integration conditions, the radiated power in (10) can be rewritten as:

(11) $$P_{Rct,n_{p}^{\eta }}\cdot {\left(\frac{R}{R_{c,n_{p}^{\eta }}}\right)}^{4}\leq P_{t,n_{p}^{\eta }}\leq P_{Rct}\cdot {\left(\frac{R}{R_{c,n_{p}^{\eta }}}\right)}^{2}$$

where, $R_{c,n_{p}^{\eta }}={\left(\frac{\sigma }{4\pi }\cdot \frac{S_{I}}{S_{R}}n_{p}^{\eta }\right)}^{\frac{1}{2}}$, $n_{p}$ is the number of pulses, η is the coherent efficiency and η = 1 means exactly the same. In theory, when (11) exists, the airborne radar might be in a LPD state because the detection probability of the opposite PDS to the radar signal is not greater than the detection probability of the radar to the target echo. However, in real scenario, the equality of right side of (11) is a critical LPD state which could not be suitable to meet the actual requirement. Therefore, the LPD design is to make the right side of (11) is far away from $P_{t,n_{p}^{\eta }}$ as much as possible when (11) exists. Similar to (10), the solution of $P_{t,n_{p}^{\eta }}$ and $n_{p}$ in (11) are also not always available which would be explained later by analyzing the value of $n_{p}$. The important constraint hidden in Formula (11) is to maintain its detection probability under a certain false alarm probability, which is defined as:

(12) $$P_{d}=\int_{U_{T}}^{\infty }\frac{r}{\sigma_{n}^{2}}\exp\left(-\frac{r^{2}+A^{2}}{2\sigma_{n}^{2}}\right)I_{0}\left(\frac{rA}{\sigma_{n}^{2}}\right)dr$$

where, $U_{T}=\sqrt{2\sigma_{n}^{2}\ln\left(1/P_{fa}\right)}$ is the detection level, $I_{0}\left(\cdot \right)$ is a zero-order Bessel function, $\sigma_{n}$ is a noise standard deviation, and $P_{fa}$ is a false alarm probability. *A* is the amplitude of the interference and *r* is the amplitude of the signal.

As can be seen from Equations (10) and (11), a possible approach is to change $S_{I}$ in Equation (7) to keep the airborne radar in LPD state. However, the general sensitivity of an advanced PDS is approximately −80 dBm, so common LPD methods such as minimizing radiation power, dwell time and maximum tracking interval are not suitable against an advanced PDS. When those common LPD methods become invalid, Equation (11) is not existed and is not again suitable to describe the LPD state of the airborne radar. However, the detection probability in (12) is still useful to describe LPD state of the airborne radar. To make the airborne radar be in LPD state, in most time, the detection probability of the airborne radar is often demanded to be greater than or equal to 0.8 while the detection probability of the opposite PDS is often demanded to be less than or equal to 0.2.

Noise interference is an alternative method of reducing the sensitivity of passive detection systems. In theory, advanced air-to-air missiles guided by the PDS could threaten airborne radar about 90 km away. According to (2) and (3), there is no solution in (11) if the airborne radar tries to detect the target 90 km away without being detected by the PDS on target when σ = 1 m${}^{2}$, $S_{R}=-$110 dBm, $S_{I}=-80$ dBm, η = 1 and $n_{p}\leq 10^{7}$.

For a maneuvering target, the number of pulse of airborne radar signal is not possible dwell on target over $10^{7}$ which is about 20 s at least if the pulse width is 1 μs and the duty cycle is 50%, so the interference is necessary to keep the airborne radar in LPD state. However, in order to protect the platform of the airborne radar, it is still necessary to control the radiation power of interference to make the interference effective without jamming the target tracking process. In complex confrontation scenarios, the PDS always uses a constant false alarm rate (CFAR) to avoid unacceptable false alarms. Although some novel CFAR methods have been proposed, the commonly used but effective CFAR algorithms for real time PDS are CA-CFAR, GO-CFAR, SO-CFAR and OS-CFAR. In order to interfere with the opposed PDS in the target tracking process, this paper proposes an adaptive radiation power control method of the airborne jammer based on the predicted radiation power of the airborne radar, and shows that the time synchronization performance of the airborne jammer based on the radiation time of the airborne radar is necessary to avoid the target tracking process being interfered by the airborne jammer.

## 3. Adaptive Radiation Power of Airborne Jammer

### 3.1. Detection Probability of CA-CFAR in Jamming

As for the average (mean level, ML) monopulse CFAR detector, let $x_{i}\left(i=1,\cdots ,n\right)$ and $y_{i}\left(i=1,\cdots ,n\right)$ represent the reference units (also referred to as the front and the rear along the reference sliding window) on both sides of the detection unit respectively. Let the reference length of sliding window be R=2n, and let the detection unit be close to the two protection units so as to avoid the leakage of the target energy into the reference units and affect the estimation of the clutter intensity. The adaptive decision criterion is:

(13) $$\begin{array}{c} H_{1}:D\geq a\sigma_{n}^{2}\\ H_{0}:D<a\sigma_{n}^{2} \end{array}$$

where, $H_{0}$ represents the assumption that there is no object. $H_{1}$ represents the assumption that the target exists. $\sigma_{n}^{2}$ is the estimation of the interference power level in the reference sliding window, the *a* is the nominal factor, and *D* is the detection statistic in the detection unit. The received clutter obeys Gaussian distribution.

In the CA-CFAR detector, the estimation of the background clutter power level is the average of 2n reference units, which is the maximum likelihood estimation of the clutter power level given that the reference units samples obeys the exponential distribution [23,24]. Define:

(14) Z=X+Y

where, *Z* is the total clutter power level estimation. $X=\sum_{i=1}^{n}x_{i}$, $Y=\sum_{i=1}^{n}y_{i}$, which are independent random variables.

(15) $$f_{D}\left(x\right)=\frac{1}{\sigma_{n}^{2}}\exp\left[-\frac{z}{\sigma_{n}^{2}}\right],x\geq 0$$

(16) $$P_{d,CA}={\left(1+\frac{a}{1+\lambda }\right)}^{-2n}$$

Among them, *a* is a nominal factor, where, λ is the predicted SNR on PDS according to predicted radiation power of the airborne radar at the next time, and the relationship between the false alarm probability and the nominal factor *a* is:

(17) $$a={\left(P_{fa,CA}\right)}^{-\frac{1}{2n}}-1$$

From (17), we can see that the detection probability and the false alarm probability have nothing to do with the average noise. Therefore, CA-CFAR has CFAR characteristics.

Assuming that there is interference in the reference cell and the interference power is $\gamma_{J}$. From (13), the decision criterion with interference becomes $a\left(1+\frac{\gamma_{J}}{2n\sigma^{2}}\right)\sigma^{2}$ and Equation (16) can be written as:

(18) $$P_{d,CA}={\left(1+\frac{a(1+\frac{\gamma_{J}}{2n\sigma^{2}})}{1+\lambda }\right)}^{-2n}$$

If the purpose of interference is to control the probability from $P_{d,CA}$ to $P_{d0,CA}$, there is:

(19) $$\gamma_{J}=2n\sigma^{2}{\left(\frac{(P_{d0,CA}^{-\frac{1}{2n}}-1)1+\lambda }{a}-1\right)}^{-2n}$$

### 3.2. Detection Probability of GO-, SO-, OS-CFAR in Jamming

The false alarm probability detected by CA-CFAR will increase at the clutter edge, and if the radar signal appear in the sliding window, the detection performance of the detector will be reduced. As a modification scheme of CA-CFAR, the maximum value selection of CFAR detection and the minimum value selection CFAR are proposed [23,24,25]. When the interference source only exists in the front sliding window or the rear sliding window, SO-CFAR is better to detect multiple radar signals, but its false alarm capability is poor. GO-CFAR can maintain a stable false alarm probability in the clutter edge environment, but the detection performance in a multiple radar signals environment is worse. GO-CFAR is mainly used for clutter edges, which takes advantage of the maximum local estimation as the total clutter power level of the detector, that is [23,24]:

(20) $$Z=\max\left(X,Y\right)$$

(21) $$P_{fa,GO}=2{\left(1+a\right)}^{-n}-2\sum_{i=0}^{n-1}\left(\begin{array}{c} n+i-1\\ \;\;i \end{array}\right){\left(2+a\right)}^{-\left(n+i\right)}$$

(22) $$P_{d,GO}=2{\left(1+\frac{a}{1+\lambda }\right)}^{-n}-2\sum_{i=0}^{n-1}\left(\begin{array}{c} n+i-1\\ \;\;i \end{array}\right){\left(2+\frac{a}{1+\lambda }\right)}^{-\left(n+i\right)}$$

When there are multiple interference sources, it is necessary to reduce the influence of adjacent interference sources. SO-CFAR detection uses a smaller local estimation as the total clutter power level estimation [23,24].

(23) $$Z=\min\left(X,Y\right)$$

(24) $$P_{fa,SO}=2\sum_{i=0}^{n-1}\left(\begin{array}{c} n+i-1\\ \;\;i \end{array}\right){\left(2+a\right)}^{-\left(n+i\right)}$$

(25) $$P_{d,SO}=2\sum_{i=0}^{n-1}\left(\begin{array}{c} n+i-1\\ \;\;i \end{array}\right){\left(2+\frac{a}{1+\lambda }\right)}^{-\left(n+i\right)}$$

The OS-CFAR detector is a sort of reference units samples from small to large. In a uniform clutter background, the probability density function of the *k* sample in the 2n sample is [23,24]:

(26) $$f_{k}\left(x\right)=k\left(\begin{array}{c} 2n\\ k \end{array}\right){\left[1-F\left(x\right)\right]}^{2n-k}{\left[F\left(x\right)\right]}^{k-1}f\left(x\right)$$

(27) $$F_{k}\left(x\right)=\sum_{i=k}^{2n}\left(\begin{array}{c} 2n\\ \;i \end{array}\right){\left[1-F\left(x\right)\right]}^{2n-i}{\left[F\left(x\right)\right]}^{k}$$

where the samples of the detection units are $x_{i}\left(i=1,2,\cdots ,2n\right)$. The OS-CFAR detector first sorts the reference units samples in ascending order.

(28) $$x_{\left(1\right)}\leq x_{\left(2\right)}\leq \cdots \leq x_{\left(2n\right)}$$

(29) $$f_{z}\left(x\right)=\frac{k}{\mu }\left(\frac{2n}{k}\right)e^{-\left(2n-k+1\right)\frac{z}{\mu }}{\left(1-e^{-\frac{z}{\mu }}\right)}^{k-1}$$

The probability and false alarm probability of the OS-CFAR detector in a uniform clutter background are:

(30) $$P_{d,OS}=k\left(\begin{array}{c} 2n\\ k \end{array}\right)\frac{\Gamma \left(2n-k+1+\frac{a}{1+\lambda }\right)\Gamma \left(k\right)}{\Gamma \left(2n+1+\frac{a}{1+\lambda }\right)}$$

(31) $$P_{fa,OS}=k\left(\begin{array}{c} 2n\\ k \end{array}\right)\frac{\Gamma \left(2n-k+1+a\right)\Gamma \left(k\right)}{\Gamma \left(2n+1+a\right)}$$

From (17), (21), (24) and (31), *a* is related to false alarm rate and the number of reference cell. However, the key point of this paper is to limit the probability of detection so that this paper only takes (16), (22), (25) and (30) into account. From (22) and (25), their average detection probability is:

(32) $$P_{d,GO}+P_{d,SO}=2{\left(1+\frac{a}{1+\lambda }\right)}^{-n}>2P_{d,CA}$$

which illustrates that interference power on $P_{d,GO}$ or $P_{d,SO}$ must be greater than that on $P_{d,CA}$. And $\max\left(\gamma_{J,GO},\gamma_{J,SO}\right)>\frac{\gamma_{J,CA}}{2n}$, where $\gamma_{J,CA}$ is defined in (19).

As for (30), with $\Gamma \left(s+1\right)=s\Gamma \left(s\right)$, there is:

(33) $$\begin{array}{cc} P_{d,OS} & =k\frac{2n!}{k!(2n-k)!}\frac{\Gamma \left(2n-k+1+\frac{a}{1+\lambda }\right)k!}{\prod_{i=0}^{k-1}\left(2n-i+1+\frac{a}{1+\lambda }\right)\Gamma \left(2n-k+1+\frac{a}{1+\lambda }\right)}\\ & =k\frac{\prod_{i=0}^{k-1}\left(2n-i\right)}{\prod_{i=0}^{k-1}\left(2n-i+1+\frac{a}{1+\lambda }\right)} \end{array}$$

When k=1, there is:

(34) $$P_{d1,OS}=\frac{2n}{2n+1+\frac{a}{1+\lambda }}$$

When k=2n, there is $P_{d2n,OS}=2n\frac{\prod_{i=0}^{2n-1}\left(2n-i\right)}{\prod_{i=0}^{2n-1}\left(2n-i+1+\frac{a}{1+\lambda }\right)}=2n\prod_{i=1}^{2n}\left(\frac{i}{i+1+\frac{a}{1+\lambda }}\right)$, and

(35) $$\begin{array}{cc} P_{d2n,OS}/P_{d1,OS} & =2n\prod_{i=1}^{2n}\left(\frac{i}{i+1+\frac{a}{1+\lambda }}\right)/\frac{2n}{2n+1+\frac{a}{1+\lambda }}\;\\ & =2n\prod_{i=1}^{2n-1}\left(\frac{i}{i+1+\frac{a}{1+\lambda }}\right)=2n\frac{2n-1}{2n+\frac{a}{1+\lambda }}\prod_{i=1}^{2n-2}\left(\frac{i}{i+1+\frac{a}{1+\lambda }}\right)\\ & <\left(2n-1\right)\prod_{i=1}^{2n-2}\left(\frac{i}{i+1+\frac{a}{1+\lambda }}\right)<\;\cdots <2\left(\frac{1}{2+\frac{a}{1+\lambda }}\right)<1 \end{array}$$

which means that the detection probability of OS-CFAR decreases when *k* increases.

From (22) and (25), there is $P_{d,GO,n}=2{\left(1+\frac{a}{1+\lambda }\right)}^{-n}-P_{d,SO,n}>0$. With *n* increases, ${\left(1+\frac{a}{1+\lambda }\right)}^{-n}$ decreases so that $P_{d,SO,n}$ must decrease to keep $P_{d,GO,n}=2{\left(1+\frac{a}{1+\lambda }\right)}^{-n}-P_{d,SO,n}>0$. From (25) and (34), we know that when n=1, there is $P_{d,SO}>P_{d1,OS}$. When *n* is sufficiently large, $P_{d1,OS}\to 1$ when $P_{d,SO,n}$ decreases according to the analysis of (22), (25) and (34).

That illustrates that the interference power of blocking CA, GO, SO and OS-CFAR only needs to interfere GO, SO and OS-CFAR. That is $\gamma_{J}=\max\left(\gamma_{J,GO},\gamma_{J,SO},\gamma_{J,OS}\right)>\frac{\gamma_{J,CA}}{2n}$, when the interference is evenly distributed in the reference cell.

## 4. Track while Jamming Design

**Figure 2:**
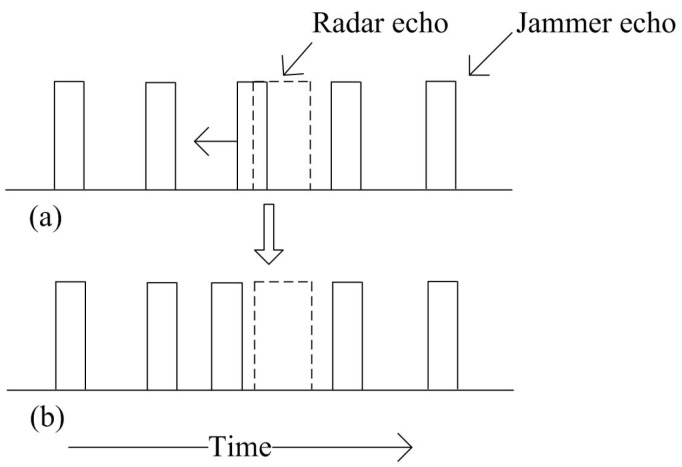
Relationship between radar echo and jammer echo.

From Figure 2, the target tracking process would be interfered if the radar echo and jammer echo is overlapped showed in Figure 2a. Since that, the time synchronization performance is very important to separate radar echo and jammer echo showed in Figure 2b. Therefore, an interference model based on adaptive radiation power design is proposed, which can not only maintain the target tracking performance, but also jam PDS on target without considering the CFAR modes.

(36) $$\begin{array}{c} \min\;E_{j}=\sum_{i}A_{j}^{i}\tau_{j}^{i}\\ s.t\;\;\;\left\{\begin{array}{c} P_{d,CFAR}\leq 0.2\\ P_{d}\geq 0.8\\ A_{j}^{i}\geq 0\\ \left(t_{j},t_{j}+\tau_{j}\right)\cap \left(t_{r},t_{r}+\tau \right)=⌀ \end{array}\right. \end{array}$$

where $E_{j}$ is the total interference energy, $P_{d,CFAR}$ is the maximum detection probability of opposite PDS supposing that its CFAR modes might include CA, GO, SO, and OS-CFAR, $P_{d}$ is the detection probability of airborne radar, $A_{j}^{i}$ and $\tau_{j}^{i}$ represent the interference amplitude and interference time of the *i*th illumination time of airborne radar respectively, $t_{j}$ is the arrival time of jammer echo at radar receiver and $\tau_{j}$ is the pulse width of jammer echo, $t_{r}$ is the arrival time of radar echo at radar receiver and τ is the pulse width of radar echo. As mentioned in Section 2, let $P_{d,CFAR}$ be less than or equal to 0.2, and let $P_{d}$ be greater than or equal to 0.8. In (36), the predicted radiation power of the airborne radar is subject to $P_{d}$, the radiation power of the jammer is subject to $P_{d,CFAR}$ and the predicted radiation power of the airborne radar. In addition, what we should pay attention to is that the predicted radiation power of the airborne radar is not constrained by (11) because (11) is existed only in non-interference conditions.

## 5. Simulations

As for the simulation scene, we assume that the initial distance between the aircraft and the target in Figure 1 is 180 km, and their initial relative speed is 280 m/s which is a general subsonic speed so that the Doppler filter algorithm and other non-coherent detection algorithms for subsonic speed target tracking are all suitable. As for the key parameters of radar signal, this paper assumes that the pulse width and the duty cycle of radar signal are 1 μs and 10% respectively. And some other parameters of (2) are shown in Table 1. As for the opposite PDS on target, this paper assumes that the width of reference unit of the CFAR detector of PDS is 1 μs, and the length of the reference units include three modes which are 6, 8 and 12 respectively.

**Figure 3:**
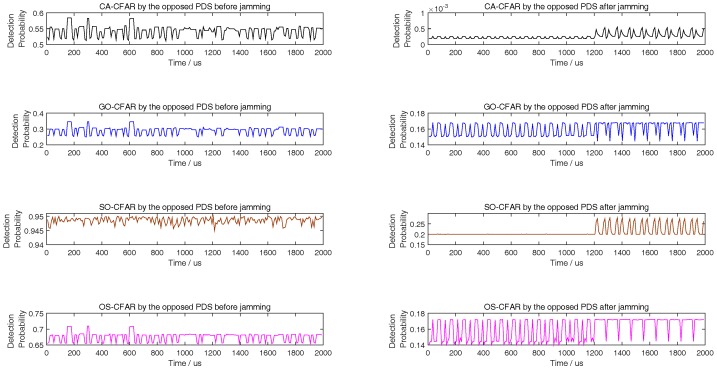
Detection probability of CA, GO, SO, and OS-CFAR before and after jamming when the number of reference units is 6.

**Figure 4:**
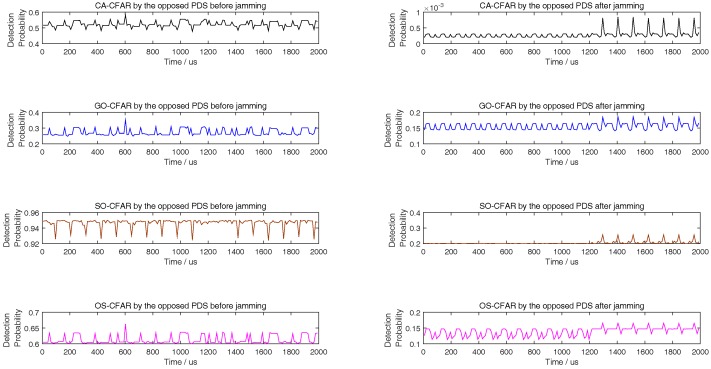
Detection probability of CA, GO, SO, and OS-CFAR before and after jamming when the number of reference units is 8.

**Figure 5:**
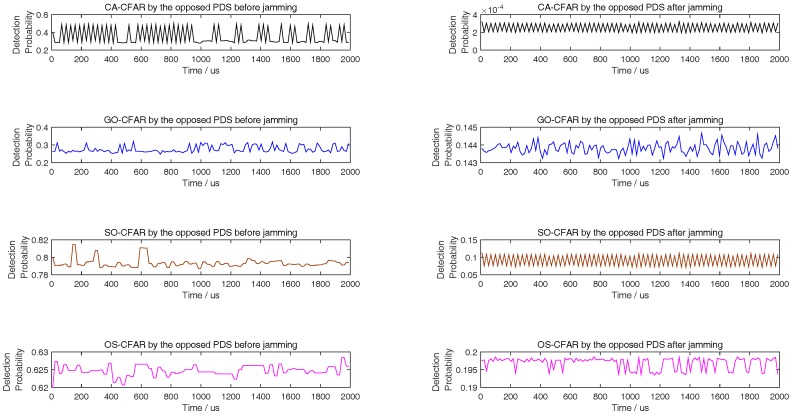
Detection probability of CA, GO, SO, and OS-CFAR before and after jamming when the number of reference units is 12.

**Figure 6:**
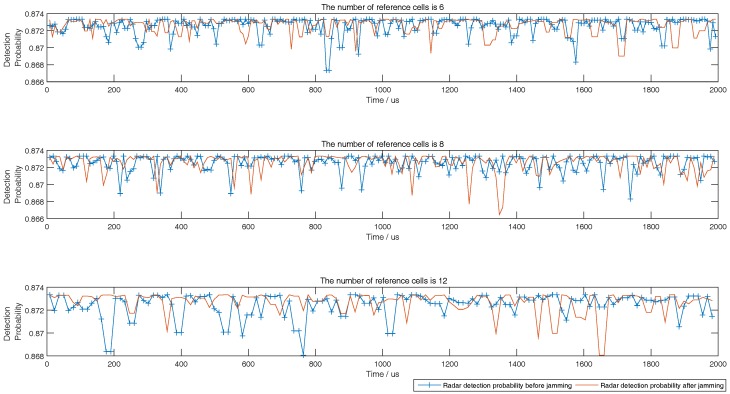
Detection probability of airborne radar after jamming.

**Figure 7:**
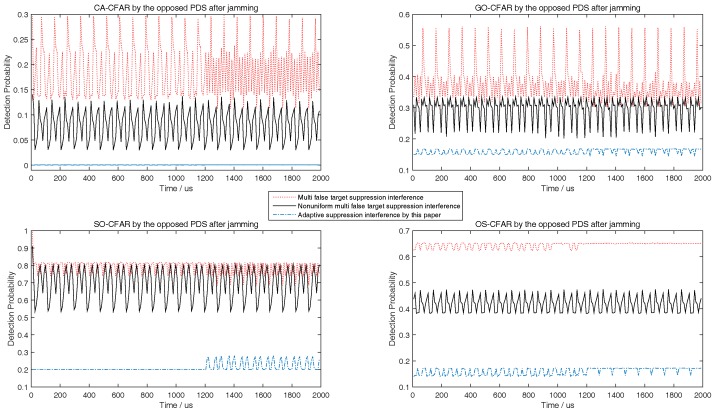
Comparing to [31,32], detection probability of CA, GO, SO, and OS-CFAR before and after jamming when the number of reference units is 6.

**Figure 8:**
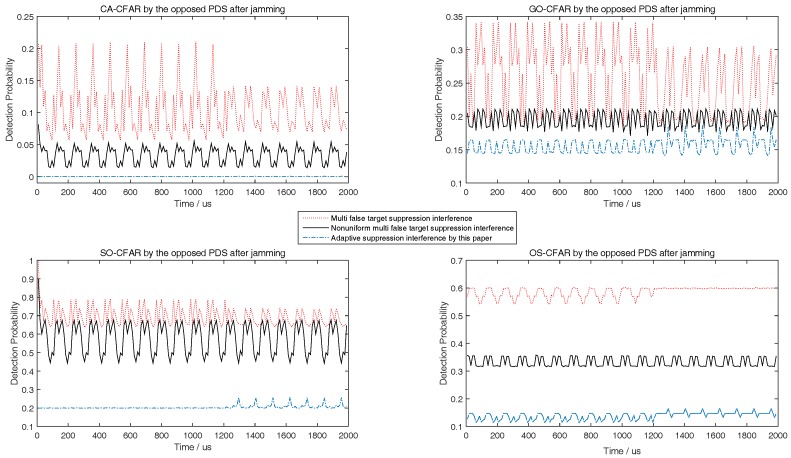
Compared to [31,32], detection probability of CA, GO, SO, and OS-CFAR before and after jamming when the number of reference units is 8.

**Figure 9:**
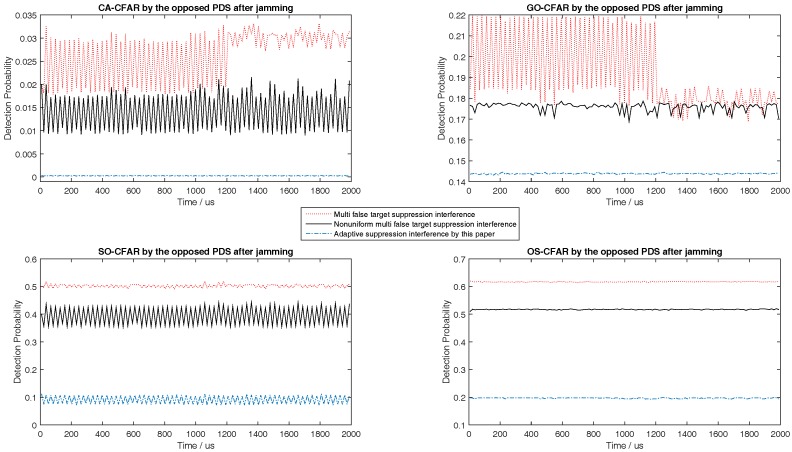
Compared to [31,32], detection probability of CA, GO, SO, and OS-CFAR before and after jamming when the number of reference units is 12.

**Figure 10:**
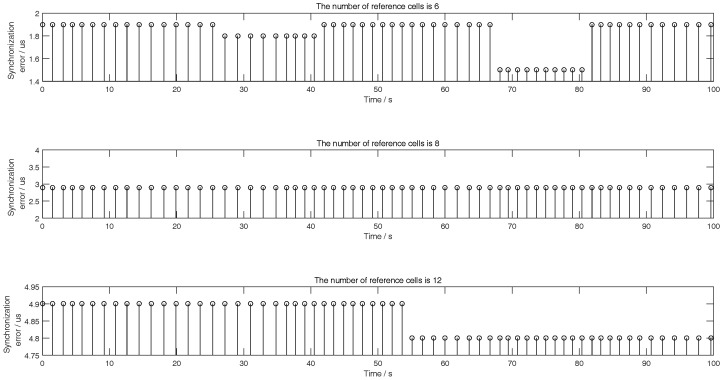
Acceptable synchronization error for different reference cells during tracking process.

Although the target echoes process method by the airborne radar is not the contribution of this paper, the $SNR_{min}$ in Table 1 must be satisfied in simulation to keep the detection probability of the airborne radar to be greater than or equal to 0.8. According to the determined $SNR_{min}$, the predicted minimum radiation power of airborne radar should meet $SNR_{min}$ as close as possible. Then, the corresponding minimum interference power of jammer related to the predicted minimum radiation power of airborne radar should meet the upper limit of $P_{d,CFAR}$ in (36) as close as possible. This principle would be used in the following simulations from Figure 3, Figure 4, Figure 5, Figure 6, Figure 7, Figure 8, Figure 9 and Figure 10.

During the target tracking process, with the general interactive multiple models Kalman filter (IMMKF) and adaptive sampling by the airborne radar [30], the state equations of maneuvering target in simulations is showed in Table 2, in which there are three models $F_{1}$, $F_{2}$, $F_{3}$, and $\Gamma_{1}=sin\left(0.05T\right)/0.05$, $\Gamma_{2}=\left[cos\left(0.05T\right)-1\right]/0.05$, $\Gamma_{3}=cos\left(0.05T\right)$, $\Gamma_{4}=sin\left(0.05T\right)$. And the threshold of tracking accuracy is set to 160 m, and *T* is set to 0.1. Obviously, the target speed is higher; the sampling interval by airborne radar is smaller. Since the target tracking process is not the key point of this paper, the simulation here assumes that the target RCS is a constant and would not show the target tracking process. To illustrate the tactic of this paper, this paper at first simulates the detection performance of different CFAR when airborne jammer is invalid, and simulates the interference results when radar echo exists or not according to (36) in Figure 3, Figure 4 and Figure 5. Then, to show that the jammer echo does not interfere the tracking process, this paper compares the detection probabilities at each tracking time with and without interference process. Finally, through comparing the interference results with other two methods, this paper shows that the method proposed by this paper is effective.

The left figures of Figure 3, Figure 4 and Figure 5 show the detection performance of different CFAR with different length of reference units, which illustrate that the detection performance of SO-CFAR is better when the length of reference units are 6 and 8. However, when the length of reference units is 12 the detection performance of OS-CFAR is better. According to (36), when $P_{d,CFAR}\leq 0.2$ and without radar echo, the simulation results in the right figures of Figure 3, Figure 4 and Figure 5 show that the interference pulse has to fill in 4, 6 and 4 reference units of CFAR detector of the opposite PDS whose reference units length are supposed to be 6, 8 and 12 respectively.

When the horizontal coordinate is less than 1200 and there is no radar echo, the right figures of Figure 3, Figure 4 and Figure 5 show that $P_{d,CFAR}\leq 0.2$ well according to (36). However, when the horizontal coordinate is larger than 1200 and the radar echo appears, the interference results in Figure 3, Figure 4 and Figure 5 changed rapidly because the jammer echoes have to avoid to interfere radar echoes as Figure 2b shows. Although the value of vertical coordinate changed rapidly in the right figures of Figure 3, Figure 4 and Figure 5 when the horizontal coordinate is larger than 1200, their maximum value is still less than 0.3 which illustrates that the math model proposed by this paper is still effective to stop the opposite PDS to detect airborne radar signal easily.

The right figures of Figure 3, Figure 4 and Figure 5 also show that the interference results depend on the mode and the reference length of CFAR. When the reference units are 6 and 8, the right figures of Figure 3 and Figure 4 show that the interference power is effective as long as SO-CFAR is interfered. However, when the reference units is 12, the right figures of Figure 5 shows that the interference power is effective only OS-CFAR is interfered. Figure 3, Figure 4 and Figure 5 also show that the conclusion that CA-CFAR is susceptible to interference in the last paragraph of Section 3 is correct.

Figure 6 compares the detection probabilities of airborne radar before and after interference by airborne jammer, which shows that the detection probabilities of airborne radar are almost higher than 0.8 constrained by (36). From Figure 3, Figure 4, Figure 5 and Figure 6, the airborne jammer is useful to interfere the opposite PDS but almost does nothing to airborne radar.

Although there are many smart interference tactics in literatures, noise interference is always the simple but very effective tactic. In addition, the smart interference frequently is only useful in special scene. To illustrate that some smart interference tactics are invalid in the simulation scene of this paper, we take the multiple false target interference and the non-uniform false target interference [31,32] into comparison. From Figure 7, Figure 8 and Figure 9, with different length of reference units, the simulation results show that the noise interference according to (36) is the simple but most effective tactic.

From (36), there is a necessary constraint $\left(t_{j},t_{j}+\tau_{j}\right)\cap \left(t_{r},t_{r}+\tau \right)=⌀$ which is the key point to maintain the detection probability of airborne radar showed in Figure 6. In fact, $\left(t_{j},t_{j}+\tau_{j}\right)\cap \;\left(t_{r},t_{r}+\tau \right)=⌀$ is influenced by the time synchronization performance. From Figure 3, Figure 4, Figure 5, Figure 6, Figure 7, Figure 8 and Figure 9, this paper assumes that the time synchronization performance of airborne radar and airborne jammer is perfect. However, the time synchronization error is always exists. The simulation in Figure 10 is to show the acceptable synchronization errors for different lengths of reference units. Figure 10 indicates that the shorter the reference units is, the smaller the acceptable synchronization error is. From Figure 10, we take the minimum value as the acceptable synchronization error of tracking while jamming system because the number of reference cells of the opposite PDS is difficult to know in advance, so that the acceptable synchronization error is 1.5 μs which is showed in the top subgraph. Although the simulated acceptable synchronization error in the middle subgraph is a constant, this is only a coincidence which might be from the assumed simulation parameters above and the minimum step of synchronization error which is 0.1 μs in simulation.

## 6. Conclusions

The opposite advanced PDS makes it diffcult for conventional methods (such as minimizing the radiation power) to maintain the airborne radar in the LPD state. This paper argues that another way to keep the airborne radar in the LPD state is to interfere with the opposite PDS on the target while the airborne radar is tracking the target. Through analysis and simulations, we illustrate that our tactic is effective when the interference power and time synchronization performance of airborne jammer meet the necessary requirements.

## Figures and Tables

**Table 1:** Parameters of the airborne radar.

Radar	Values	Radar	Values	Radar	Values
$G_{t}$	30 dB	$G_{rr}$	30 dB	σ	1 m${}^{2}$
*L*	3 dB	$B_{r}$	1 MHz	$F_{n}$	2 dB
${SNR}_{min}$	15 dB	τ	1 μs	$T_{dmax}$	10 ms
$P_{t}^{\max}$	20 kw	$P_{t}^{\min}$	10 w	λ	0.03 m

**Table 2:** Maneuvering process of target.

Time(s)	State Equations
0∼29	$F_{1}^{T}=\left[1,T,0,0;0,1,0,0;0,0,1,T;0,0,0,1\right]$
30∼50	$F_{2}^{T}=\left[1,\Gamma_{1},0,\Gamma_{2};0,\Gamma_{3},0,\Gamma_{4};0,-\Gamma_{2},1,\Gamma_{1};0,-\Gamma_{1},0,\Gamma_{3}\right]$
50∼59	$F_{1}^{T}=\left[1,T,0,0;0,1,0,0;0,0,1,T;0,0,0,1\right]$
60∼80	$F_{3}^{T}=\left[1,-\Gamma_{1},0,\Gamma_{2};0,\Gamma_{3},0,-\Gamma_{4};0,-\Gamma_{2},1,-\Gamma_{1};0,\Gamma_{1},0,\Gamma_{3}\right]$
80∼end	$F_{1}^{T}=\left[1,T,0,0;0,1,0,0;0,0,1,T;0,0,0,1\right]$
